# Human social genomics: Concepts, mechanisms, and implications for health

**DOI:** 10.1002/lim2.75

**Published:** 2023-02-25

**Authors:** George M. Slavich, Summer Mengelkoch, Steven W. Cole

**Affiliations:** 1 Department of Psychiatry and Biobehavioral Sciences, University of California, Los Angeles, California, USA; 2 Department of Medicine, University of California, Los Angeles, California, USA

**Keywords:** behavior, disease, health, longevity, resilience, social experiences, stress

## Abstract

The exciting field of human social genomics provides an evolutionarily informed, multilevel framework for understanding how positive and negative social–environmental experiences affect the genome to impact lifelong health, well-being, behavior, and longevity. In this review, we first summarize common patterns of socially influenced changes in the expression of pro-inflammatory and antiviral immune response genes (e.g., the Conserved Transcriptional Response to Adversity), and the multilevel psychological, neural, and cell signaling pathways by which social factors regulate human gene expression. Second, we examine how these effects are moderated by genetic polymorphisms and the specific types of social–environmental experiences that most strongly affect gene expression and health. Third, we identify positive psychosocial experiences and interventions that have been found to impact gene expression. Finally, we discuss promising opportunities for future research on this topic and how health care providers can use this information to improve patient health and well-being.

## 1 | HUMAN SOCIAL GENOMICS: CONCEPTS, MECHANISMS, AND IMPLICATIONS FOR HEALTH

Decades of research has found that individuals living in disadvantaged environments are at greater risk for numerous negative health outcomes, particularly chronic diseases driven by inflammation such as cardiovascular disease, several cancers, and metabolic disorders.^1–6^ Despite modern scientific insights into mechanisms underlying these diseases, rates of chronic disease are rising throughout even the most economically well-off nations.^7^ One explanation for this finding lies in people’s inherent drive to achieve a relatively advantaged status.^6,8^ Indeed, despite being well-off relative to millions of other people worldwide, individuals living in poverty or in conditions of low socioeconomic status (SES) in a First World country can experience high levels of social stress due to their relatively disadvantaged social status as compared to others in their immediate environments. Rather than relative socioeconomic disadvantage simply being a financial stressor, it also constitutes a form of social adversity that drives disease-relevant biological processes.^6,9,10^

One way that social–environmental factors such as low social status “get under the skin” to impact health and behavior is through nervous system regulation of immune system components involved in inflammation. In fact, chronic, “nonresolving” inflammation is a well-known risk factor for numerous diseases of aging; moreover, it is predicted by different types of life stressors but especially by social stressors, such as social conflict, isolation, rejection, and exclusion.^11,12^ Rather than upregulating inflammatory activity in a localized and time-limited manner such as what occurs when you get a papercut, social stressors have the ability to set in motion a complex set of psychological and biological changes that can lead to systemic chronic inflammation; additionally, they alter how the body responds to both psychosocial stressors and immunological threats.^10^ One mechanism by which these effects occur is through social stress-related epigenetic changes in the function and expression of immune response genes.^13,14^ For example, a growing body of research has shown that experiencing early-life social stressors can alter the gene expression of children in a way that promotes the development of a pro-inflammatory phenotype, which has the ability to persist into adulthood even in the absence of ongoing stress.^15–22^ These epigenetic changes can, in turn, substantially affect individuals’ mental and physical health, as well as their overall well-being and mortality risk.^14^

In the following sections, we provide a general overview of the emerging field of human social genomics with a focus on its basic concepts, mechanisms, and implications for health. First, we discuss the basic mechanisms of human gene expression, followed by a discussion of some ways in which social factors can impact those processes. Second, we highlight one common pattern of socially influenced changes in gene expression, known as the Conserved Transcriptional Response to Adversity (CTRA), which is characterized by the increased expression of pro-inflammatory and reduced expression of antiviral immune response genes as a function of experiencing social adversity. Third, we turn our attention to the mechanisms by which these social factors are translated into biochemical processes that directly control gene transcription and, in addition, how this “social signal transduction” is moderated by genetic polymorphisms and other individual difference factors. Fourth, we discuss how specific types of social–environmental stress and adversity can impact gene expression in ways that lead to poor health outcomes. Fifth, we highlight positive psychosocial experiences and interventions that have been found to impact gene expression in ways that can lead to better health and well-being. Finally, we discuss several promising foci for future research on this topic and propose some first steps that health care providers can take to leverage their understanding of human social genomics to improve the prevention, diagnosis, and treatment of disease.

## 2 | HUMAN GENE EXPRESSION

Although humans can readily detect changes in thoughts and emotions in response to changing social circumstances, we generally experience our biological selves as being relatively stable over time. After all, our brains and bodies were built based upon our DNA, our basic genetic blueprint of approximately 20,000 genes, which is carried within each of our cells from the time we are conceived until our death and changes very little throughout our lives. Although our DNA remains relatively stable over the life course, our molecular selves are constantly changing. In fact, 1%–2% of our cells are replaced every day.^23^

Whereas in the past, scientists assumed that an aging cell would be replaced by a new cell comprised of the same protein structure used to build the old cell, advancements in functional genomics have revealed that this is often not the case. Instead, although one’s DNA provides a blueprint for all the proteins that an individual *could* produce, these proteins are not all being produced all of the time. Rather, genes that code for the production of different proteins are “turned on” and “turned off” by internal and external signals, which results in differential gene expression within an organism based upon their social–environmental conditions. Within any given cell, only about half of the DNA genome is actively expressed at any given point in time. This means that as cells regenerate, they are created based on the portions of a person’s DNA that are currently being expressed (i.e., the subset of genes that are actively being transcribed into RNA). As such, the human genome is best thought of as the blueprint for all of the possible biological selves a person *could be*, as opposed to the blueprint for a *stable biological self*.

The biochemical signaling pathways that regulate human gene expression are now also known to be influenced by both physical and social–environmental conditions. Social isolation, for example, has been related to the differential expression of hundreds of genes^24–28^ that impact immune function as well as behavioral phenotypes.^25,29^ Given that our cells have an average half-life of about 80 days and are constantly being replaced, these dynamics provide insight into how a social factor such as social isolation can get under the skin, so to speak, to influence human health and behavior for years to come.^14^ These revelations have, in turn, sparked the field of human social genomics, which has yielded exciting new insights into how a variety of positive and negative social factors are associated with changes in gene expression, which, as it turns out, often predict human health and behavior more strongly than even the genetic code with which we are endowed.

To impact downstream processes like immune function and behavior, genes have to be expressed, which requires one’s genetic code to be transcribed from DNA into RNA. This process is regulated by transcription factors, which are intracellular proteins that signal which genes should be expressed as RNA at a given time. The activity of transcription factors is controlled both endogenously within cells but also by extracellular signals such as hormones, neurotransmitters, and growth factors. When a person experiences stress, for example, the endocrine system releases glucocorticoids (i.e., cortisol), and the sympathetic nervous system releases catecholamines, such as norepinephrine. These chemicals are detected by cellular receptors that initiate a cascade of intracellular reactions, resulting in the activation of transcription factors which then bind to the “regulatory” regions of DNA that lie upstream of the protein-coding portion of a gene. Genetic polymorphisms, or individual differences in one’s DNA, can influence whether and how strongly these transcription factors bind and stimulate RNA transcription. However, generally, these activated transcription factors result in the production of proteins that influence bodily processes, impacting immune function, metabolism, and other physiological processes, as well as mood, cognition, behavior, and health. Stress-related signaling molecules such as cortisol or norepinephrine can impact transcription factor activity, and thereby affect patterns of transcriptional activity across the whole human genome (for a review, see Ref. 14).

Gene expression differs across time, cell types, and tissues, and the genes expressed in specific groups of cells at a given time are called the transcriptome. A person’s basal transcriptome is influenced by both their DNA genetic sequence and the current physiochemical environment in their body.^30,31^ Central to the field of human social genomics is the discovery that one’s social–environmental conditions, and especially their social–cognitive representations of these conditions, can trigger changes in transcriptional activity and the basal transcriptome through neural and endocrine processes that originate in the central nervous system.^32–36^

## 3 | SOCIALLY SENSITIVE GENES

Much early scientific debate on the genetic basis of psychosocial life focused on the extent to which behavior, personality, and health are influenced by a person’s genes versus the environment. Although it is now accepted that both genetic and environmental factors affect these outcomes, researchers next asked a similar question about factors affecting human gene expression (e.g., Refs. 30, 37, 38). In an early landmark study, for example, Idaghdour and colleagues^38^ found that the difference between living in an urban versus rural environment explained 10 times more differential gene expression than did genetic differences attributable to gender and ancestry. This study was not designed to identify the environmental features most responsible for explaining variability in gene expression, but it did highlight the fact that a person’s physical environment can substantially affect gene expression.

Support for the idea that *social* features of one’s environment may also affect gene expression began to emerge around the same time from research aimed at elucidating the mechanisms by which social factors influence health, immune function, and mortality in people experiencing social isolation. Although it has long been known that social isolation increases mortality risk,^39–43^ the biological mechanisms underlying this association remained unclear. To address this issue, researchers compared the genome-wide transcriptional profiles of individuals experiencing chronic social isolation to those who were socially well-integrated, focusing on the expression of immune response genes, given the strong association between immune function and mortality risk. More specifically, researchers compared the expression of innate immune genes (i.e., the pro-inflammatory cytokine genes *IL1B*, *IL6*, *IL8*, and *TNF)*, which generally combat bacterial and other extracellular pathogen threats, to the expression of adaptive immune genes (i.e., the antiviral response genes *IFNA* and *IFNB*), which target intracellular pathogenic threats like viruses.^44–46^ In doing so, researchers found that individuals experiencing social isolation exhibited heightened expression of pro-inflammatory immune response genes and decreased expression of antiviral immune response genes.^24^ These shifts in immune response gene expression were linked to increased activity of the transcription factors, NF-*κ*B and AP-1, which upregulate inflammation; decreased activity of interferon response factors, which promote innate antiviral resistance; and decreased activity of the glucocorticoid receptor, which acts as a transcription factor to downregulate inflammation^24^ (for subsequent studies replicating and generalizing these effects, see Refs. 25–28 and 47, and for a review, see Ref. 48). Given that diseases associated with social isolation are typically characterized by elevated inflammation,^49–51^ these results provided an early indication of the molecular pathways through which social isolation can lead to greater inflammatory activity, chronic disease, and mortality risk.

Although these early functional genomic studies suggesting that social influences impact expression of immune response genes were correlational, similar results have been found in response to experimental manipulations in mammalian model organisms.^52–59^ In one study, researchers found that changes in social status altered peripheral blood mononuclear cell gene expression in female rhesus macaques.^60^ More specifically, experimentally demoting female macaques to a lower social dominance rank resulted in increased expression of immune response genes that promote inflammation, an increase in glucocorticoid resistance, and altered glucocorticoid signaling. Moreover, the researchers were able to predict the macaques’ dominance rank within their group with 80% accuracy based upon the mononuclear cell gene expression data alone. Finally, the finding indicating social stressor-related alterations in glucocorticoid signaling suggests that altered glucocorticoid functioning may play a key role in driving social threat-related changes in pro-inflammatory gene expression.^58,60^

Related research found similar, health-damaging alterations in gene expression for infant macaques raised by an inanimate surrogate mother or by age-matched peers versus those raised by their own mothers.^54^ Again, bioinformatic analyses implicated increased activity of pro-inflammatory NF-*κ*B transcription factors and decreased activity of interferon response factor transcription factors in structuring the early life adversity-related changes in the leukocyte gene expression observed.^54^ Similar results emerged from subsequent studies modeling the effects of COVID-19 pandemic-style “shelter-in-place” protocols in nonhuman primates, with macaques relocated from social housing to two weeks of individual housing again showing increased pro-inflammatory activity and reduced antiviral activity.^27^ These results are consistent with the hypothesis that social processes *cause* alterations in gene expression in primates and, importantly, these primate results mirror what has been found in humans exposed to social isolation and other forms of social–environmental adversity.

## 4 | CONSERVED TRANSCRIPTIONAL RESPONSE TO ADVERSITY

**FIGURE 1:**
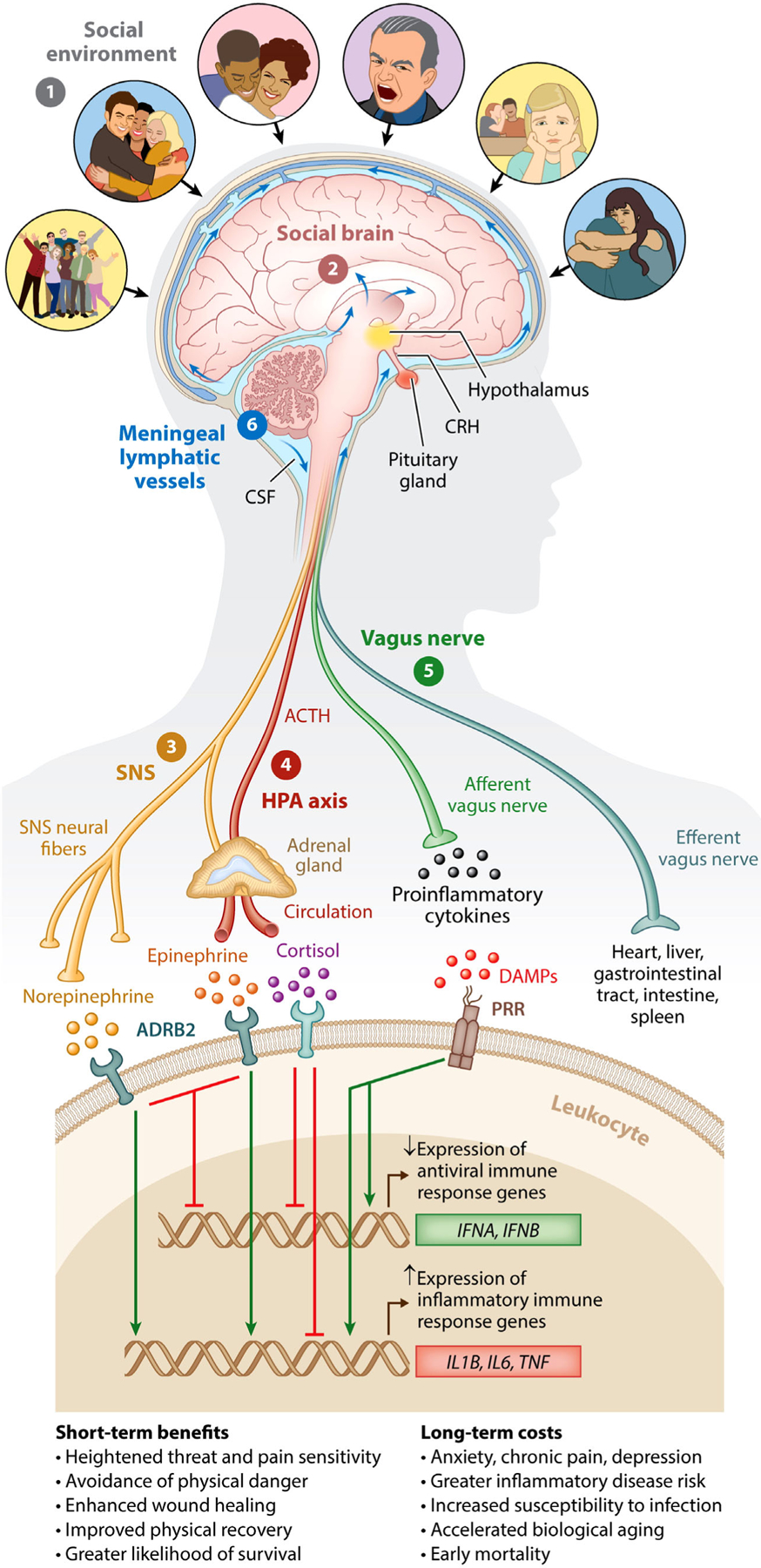
Human social signal transduction. Social signal transduction is the process by which the CNS monitors and appraises the (1) social environment, interprets social signals and behaviors, and judges the extent to which the surrounding environment is socially safe versus threatening. These appraisals are subserved by the (2) social brain. When a threat is subjectively perceived, the brain activates a multilevel response that is mediated by several potential social signal transduction pathways—namely, the (3) SNS, (4) HPA axis,(5) vagus nerve, and (6) meningeal lymphatic vessels. These pathways enable the brain to alter genome-wide transcriptional dynamics in peripheral tissues (e.g., white blood cells). In response to social adversity, the main end products of the SNS, epinephrine and norepinephrine, suppress transcription of antiviral type interferon genes (e.g., *IFNA* and *IFNB*) and upregulate transcription of pro-inflammatory immune response genes (e.g., *IL1B*, *IL6*, and *TNF*), known as the Conserved Transcriptional Response to Adversity. In contrast, the main end product of the HPA axis, cortisol, generally reduces both antiviral and inflammatory gene expression (although chronic social stress can trigger glucocorticoid insensitivity/resistance, which can allow increased inflammatory gene expression). The vagus nerve, in turn, plays a putative role in suppressing inflammatory activity, whereas meningeal lymphatic vessels enable immune mediators originating in the CNS to traffic to the periphery, where they can potentially exert systemic effects. CNS, central nervous system; HPA, hypothalamic–pituitary–adrenal; *IFNA*, interferon alpha; *IFNB*, interferon beta; *IL1B*, interleukin 1 beta; *IL6*, interleukin 6; SNS, sympathetic nervous system; *TNF*, tumor necrosis factor. Republished from Ref. 92 with permission from Annual Reviews.

The pattern of increased pro-inflammatory and decreased antiviral immune response gene expression that we have described is known as the CTRA.^14^ Figure 1 illustrates the main pathways by which the central nervous system detects social adversity and transduces these cues into neural and endocrine signals that reshape the leukocyte basal transcriptome,^32–36,61^ thus preparing the body to combat the different types of microbial exposures that have been associated with physical injury in adverse environments throughout our evolutionary past.^33,47^ The role of the central nervous system in coordinating these biobehavioral responses to threat is believed to have evolved to help to protect organisms during physical threat.^33,47,62,63^ In fact, central nervous system regulation of gene expression is vital for accelerating wound healing and preventing infection when wounding has occurred.^33,64^ In the modern social world, however, where physical wounding is rare, chronic activation of the CTRA in response to actual or imagined social stressors leads to increases in inflammatory activity and inflammation-related disease risk, alongside increased susceptibility to viral infections like the common cold due to reduced antiviral immune response gene expression.^24,27,34–36,65–68^ Paradoxically, what once served as a highly adaptive anticipatory response to impending physical threat now appears to increase our susceptibility to the types of chronic diseases that will kill nearly all of us.^69^

## 5 | SOCIAL SIGNAL TRANSDUCTION

The multilevel mechanisms by which adverse social–environmental conditions get transformed into genome-regulating biochemistry have been called social signal transduction.^12,14^ First demonstrated in nonhuman animal models, Robinson and colleagues^70^ described the key role of the central nervous system in transducing social–environmental information into neural and endocrine signals that in turn modulate gene expression in both the brain and periphery.^70–72^ The ability of social–environmental influences to remodel transcriptional activity in the brain could contribute to the mental and physical health problems that are commonly observed among those who have experienced adversity, including anxiety disorders, depression, heart disease, and autoimmune and neurodegenerative disorders.^73,74^

As depicted in Figure 1, researchers have since identified parallel processes in humans, with a person’s neurocognitive assessment of their social–environmental surroundings as being socially safe versus threatening being a key process influencing the activation of the CTRA.^63^ Indeed, in two separate lines of research, researchers have found individuals’ beliefs that their social world is threatening, hostile, unsupportive, dangerous, or inhospitable to be more strongly related to their leukocyte transcriptome than objective measures of social status, such as household income or education level, or objective measures of social connection, such as marital status or social network size.^24,47,75,76^

Collectively, these results demonstrate how it is central nervous system–mediated appraisals of social–environmental conditions that trigger the release of biochemical signals that regulate gene expression, not the social–environmental conditions themselves.^33,77,78^ Consequently, situations that have not happened yet, or that might never happen, can engage the same molecular defense programs as actual social or physical threats, simply through one’s own thoughts and imagination.^33,34,36,78^ Furthermore, the same social–environmental conditions can lead to differential gene expression in different people—and even in those with the same genotype—based upon how they appraise these situations (e.g., Ref. 79) and depending on individual differences in factors such as sensitivity to social threat,^80,81^ cognitive–emotional resources,^82^ availability of social support,^83^ and biographical and psychiatric histories.^78,84^ These findings underscore how cognitive processes and individual differences in personality and other trait–like factors can become associated with stable differ ences in basal gene expression, and, in turn, influence health over the lifespan.^24,47,85^ Finally, because people’s subjective perceptions of threat are what trigger the release of the molecules that influence gene expression, very different threats can result in the same patterns of gene expression regardless of whether they are physical (e.g., pain) or social (e.g., rejection) in nature.^78,86–88^

## 6 | TRANSCRIPTIONAL EMBEDDING OF SOCIAL EXPERIENCES

One way adverse social–environmental factors influence long-term behavior and health is through epigenetic processes. Early research in rodents revealed that poor maternal care causes epigenetic changes in the glucocorticoid receptor gene, specifically in the hypothalamus and amygdala, which is associated with heightened anxiety-like behaviors in adulthood.^89,90^ These same epigenetic modifications have also been found in humans who have experienced abuse and neglect.^91^ Together, these results provide a potential explanation for how adverse experiences early in life can promote long-lasting changes in transcriptional activity that shape complex behavioral phenotypes and risk for disease.

Although epigenetic modifications to one’s transcriptome are one way that adverse experiences can influence human behavior and disease risk, there exist several other processes through which adverse social–environmental factors early in life affect long-term behavior and health. For example, acutely stressful situations may become intensified and prolonged, similar to how a minor cold can progress into a sinus infection. Following this process, a single experience of targeted rejection in a new social group could develop into ostracism and social isolation. Additionally, an acute stressor might be transient in nature but a person’s *experience* of the stressor may become intensified or prolonged through rumination, which could prevent an individual from developing healthy social relationships due to their persistent negative thoughts about a single past negative experience or relationship (for a review of pathways, see Ref. 92).

Finally, there exist several other biological processes involved in transcriptional regulation that can generate persistent changes in biological function after an early life insult. Many gene-regulatory processes in the immune system involve feedback loops that enable an acute insult to generate a transcriptional response (e.g., production of inflammation) that can sustain itself over time, and potentially render the organism more vulnerable to a future insult (e.g., as inflamed tissue is more vulnerable to future injury). Another involves a developmental process known as biological embedding,^93–96^ which occurs when stressors are experienced during sensitive periods of development and thus shape transcriptional dynamics and the biological systems that govern these dynamics.^20,97,98^ Because our cells are constantly dying and being replaced with new cells, adverse experiences can create acute changes that become biologically embedded in our tissues. Further, because many cells such as lymphocytes live for months to years, cells generated during adverse experiences can stay with us for quite some time after an adversity has passed, giving experiences of social–environmental adversity occurring on a single day the ability to influence immune system dynamics for months or years.^33,53^ Consistent with this idea, researchers manipulated the social dominance rank of adult female macaques and found that their history of social rank, together with their current social rank, predicted their immune response gene expression, as opposed to their gene expression only being predicted by their current social status.^55^ This finding provides evidence that biological embedding can occur in adulthood, even outside of biologically sensitive periods, and in immune cells, which are typically considered to have no “memory” of past circumstances.

Beyond modifying gene expression of leukocytes, social– environmental adversity also regulates gene expression in neurons and other long-lived terminally differentiated cell types.^70,72,85^ For example, researchers have found that social instability upregulates expression of the gene that codes for nerve growth factor beta in macaque lymph nodes, which increases an organism’s vulnerability to viral infection through reduced antiviral immune response gene expression in leukocytes.^99,100^ Further, these dynamics have been shown to sensitize an organism to social–environmental influences and to potentially contribute to a feed-forward cycle that perpetuates sympathetic nervous system hyperinnervation, which can lead to ongoing subjective perceptions of increased threat.^45^ A similar neuromolecular sensitization in the central nervous system appears to occur in response to chronic stressors, such as ongoing interpersonal problems, imminent bereavement, persistent social isolation, and low SES.^25^ As a result of such transcriptional recursion, the biological residue of past adverse social–environmental conditions and experiences can become embedded in the basal cellular transcriptome and persist for years after an initial adversity has passed.^14,21,45,101^

## 7 | MODERATION BY GENETIC POLYMORPHISMS AND INDIVIDUAL DIFFERENCES

Clearly, not everyone who experiences adversity becomes ill. Although early genetic research focused on identifying genes or phenotypes that conferred specific risks versus benefits, genomic researchers have begun to turn much of their attention toward polymorphisms that influence a person’s biological sensitivity to social–environmental influences. For example, as opposed to searching for a “depression gene,” researchers focused on how certain polymorphisms may influence how strongly social–environmental influences impact inflammatory gene expression, which then affects the risk of developing depression. It seems that some alleles, called phenotypic plasticity alleles, confer an individual with advantages in favorable environments and risks in harsher environments.

These phenotypic plasticity alleles likely remained polymorphic, given the range of human evolutionary environments, because variation in a person’s sensitivity to the environment can confer both costs and benefits, depending on the environmental conditions with which one is faced.^30,45,65,66,68,102^ Although it is unclear how broadly plasticity alleles impact human health and behavior, or whether their effects remain stable across the life course, the possibility that some people might be more genetically sensitive to their social environment than others—and as such, more deeply connected to the larger network of the current human genomes surrounding them (whose own transcriptomes are also being shaped by the genomic regulation of others)—provides an intriguing avenue for future research at the intersection of culture and human social genomics. Further, because much of the genetic basis for human sensitivity to social context may lie in nonpolymorphic regions of our genome, which would not have been detected in polymorphism-based association studies, we may have yet to discover many of the genetic sequences that most strongly influence humans’ sensitivity to social–environmental experiences.^30,38,66^

Emerging evidence also indicates that individual differences in certain psychological factors may affect changes in gene expression, especially in response to stress. For example, a recent study that investigated pro-inflammatory gene expression and depressed mood following endotoxin administration in healthy participants found that perceived stress levels, trait sensitivity to social disconnection, and preexisting depressive and anxiety symptoms each moderated the association between endotoxin exposure and both depressed mood and increased activation of pro-inflammatory transcription control pathways following endotoxin exposure.^103^ Continued research aimed at uncovering psychological and biological moderators of associations between social–environmental adversity and gene expression will set the stage for a precision medicine approach to managing health outcomes associated with experiencing stress and adversity. That is, a better understanding of who is at highest risk of experiencing heightened inflammation and, in turn, poor health outcomes, resulting from adverse social–environmental conditions, will enable resources to be more appropriately tailored and deployed to help those who would benefit most, thus reducing disease burden and improving well-being for many.^104,105^

## 8 | SOCIAL FACTORS AFFECTING GENE EXPRESSION

Using a social genomics lens to understand human health and disease can be powerful. As alluded to above, social factors are well known to influence health, both positively and negatively. The social genomics approach advances this work by providing a concrete molecular framework for identifying for whom as well as why, how, and under what conditions social factors are expected to impact health outcomes through specific and targetable transcriptional pathways. For social conditions to impact gene expression in a meaningful way, for example, a person must have an environmentally sensitive genotype, and this genotype must be activated by social signal transduction processes for altered gene expression—and positive or negative health outcomes—to occur. This framework thus helps to inform why some people in the same social environments express different genes (i.e., because they have different genotypes or different appraisals of their environmental conditions) and health trajectories, as well as why individuals with the same genotypes do not share the same gene expression profiles (i.e., because they are embedded in different social–environmental conditions or have different appraisals of these conditions). The social genomics framework also enables scientists to map social signal transduction pathways to investigate ways to mitigate specific gene–environment health risks. The best understood pathways through which social conditions differently impact individual gene expression in ways that affect health outcomes are those relating to social adversity and inflammation^14,92^; however, recent research has begun to identify how many different positive and negative social experiences might impact gene expression and health. Table 1 summarizes some of the most well-established associations between social factors and human gene expression to date.

### 8.1 | Social–environmental adversity and gene expression

As we have discussed, experiencing social–environmental adversity has been associated with an increased risk for several negative health outcomes, especially those related to inflammation, including asthma, rheumatoid arthritis, cardiovascular disease, certain cancers, and depression.^106^ Some of the most compelling evidence that social–environmental factors affect gene expression comes from a landmark study that examined associations between social–environmental adversity and risk from both inflammatory and non-inflammatory related diseases. Building on data showing that a specific transcription factor, GATA1, mediated transcriptional responses to adversity through the sympathetic nervous system, Cole and colleagues^53^ identified a single nucleotide polymorphism in the sequence of a pro-inflammatory cytokine gene that computational modeling suggested might affect the binding of GATA1 and, therefore, the expression of that gene.

**FIGURE 2:**
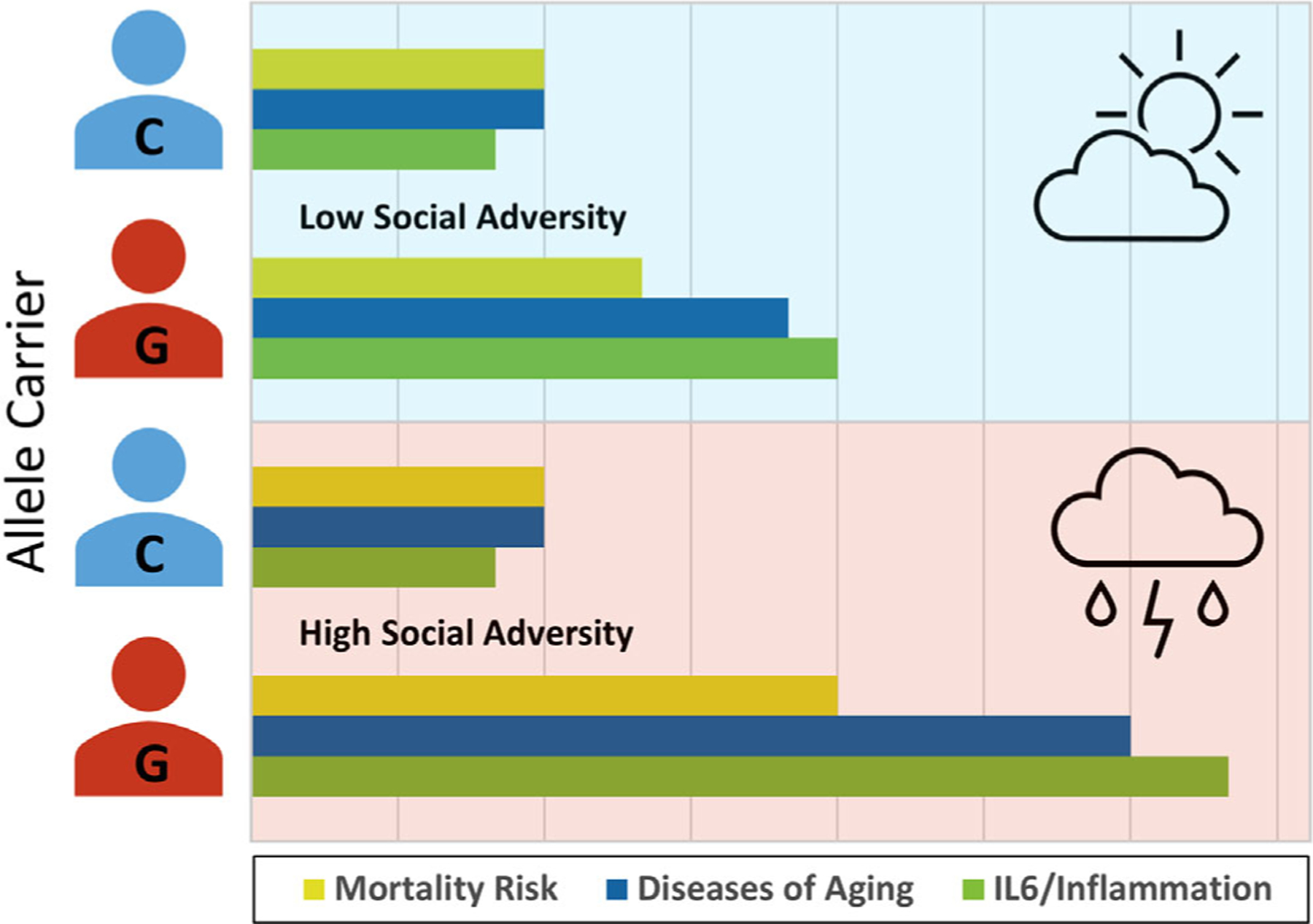
Gene–environment interactions and health. Graphically depicted are the results of the study by Cole and colleagues^53^, who found that a single nucleotide polymorphism in the human *IL6* promoter alters the likelihood of threat-activated GATA1 transcription factors to bind to DNA to stimulate *IL6* transcription. Individuals homozygous for the GATA1-sensitive G allele have high binding affinity, which enables stress-induced GATA1 activity to upregulate *IL6* gene expression, leading to greater inflammatory activity, a higher likelihood of developing diseases of aging, and elevated mortality risk in the context of social adversity. In contrast, those with the C allele at this locus have low binding affinity for GATA1, meaning that the biochemical social adversity signals do not efficiently stimulate *IL6* gene expression. As such, C allele carriers at this locus experience standard levels of inflammation, prevalence of diseases of aging, and mortality risk under both socially favorable and socially adverse conditions.

As depicted in Figure 2, these researchers found a G/C transversion 174 bases before the transcription start site of the human *IL6* gene that impacted GATA1 binding. Whereas C allele carriers in this study experienced the same inflammatory disease risk regardless of their social conditions, carriers of one or more GATA1-responsive G alleles experienced relatively greater risk of inflammatory diseases, which was further heightened in the context of social–environmental adversity. In fact, those who were homozygous for the GATA1 sensitive G allele died 2.8 years sooner under high social–environmental adversity than did their counterparts with the GATA1 insensitive C allele. These results suggest that social–environmental threats induce biochemical signals that drive inflammatory processes for G (but not C) allele carriers. Looking forward, findings like this can point to novel approaches for treating and potentially preventing inflammatory diseases, which cause substantial disease burden throughout the world.^11,107–109^

As reviewed above, experiencing social isolation is associated with altered gene expression and higher risk of morality due to inflammation-related diseases.^49–51^ Recently, many people throughout the world experienced a period of sustained social isolation caused by shelter-in-place restrictions that were enacted to decrease people’s risk of developing COVID-19.^10^ To understand how the social deprivation associated with these restrictions might affect immune function, researchers subjected adult male macaques to similar restrictions and found that, within 2 days of being isolated, shelter-in-place isolation resulted in an overall decrease in immune cells of 30%–50%, downregulation of interferon antiviral gene expression, and a relative upregulation of monocytes; moreover, these effects lasted for 2–4 weeks after being returned to social housing.^27^ Next, the researchers investigated the impact of housing the macaques with a juvenile male during the shelter-in-place isolation period. They found similar decreases in circulating immune cells; however, the macaques housed with a juvenile did not display decreased interferon gene expression or increased monocyte levels.^27^ These results thus highlight how social relationships help mitigate the negative gene regulatory effect that social isolation typically has on the body.^127^

Although supportive social relationships are associated with decreased expression of pro-inflammatory immune response genes, attempts to increase social connection can increase a person’s risk of experiencing social threats, such as targeted rejection, which can upregulate pro-inflammatory social signal transduction pathways and downregulate anti-inflammatory pathways, increasing the likelihood of developing both mental and somatic conditions, such as depression and asthma (e.g., Refs. 122, 128). In this context, Slavich and colleagues^123^ found that individuals are more likely to develop depression following a major life event characterized by targeted rejection if they carry the G allele for the *μ*-opioid receptor gene (*OPRM1*, rs1799971). Specifically, G allele carriers who experienced targeted rejection in this study were twice as likely to meet diagnostic criteria for major depressive disorder relative to A/A homozygotes, potentially due to reduced opioid receptor expression and signaling efficiency after experiencing social pain inherent in targeted rejection. Indeed, past research has found that this SNP causes an amino acid change (N40D) that affects *OPRM1* expression, resulting in differences in sensitivity to social and physical pain. Conversely, Slavich and colleagues^129^ further showed that damping neural pain signaling with acetaminophen may alleviate daily experiences of social pain, particularly for highly forgiving individuals. Results such as these highlight that even time-limited experiences of social threat can alter the leukocyte basal transcriptome—effects that can possibly be mitigated by the presence of positive social relationships and interventions that reduce the activity of social signal transduction pathways that drive inflammation.

Once positive, biologically beneficial social relationships can also become a source of social–environmental threat. For instance, when a person becomes chronically ill, caregiving duties often fall to those closest to them. Caregiving for a critically ill loved one is stressful. In fact, even anticipating the loss of a spouse can alter the leukocyte basal transcriptome.^67^ Studies that have investigated the gene expression patterns of caregivers have found that relative to control participants, caregivers demonstrate evidence of the CTRA. For example, caregivers exhibit decreased expression of genes with response elements for the glucocorticoid receptor, which can reduce inflammation, and increased inflammatory gene expression, as compared to control participants.^124^ These data highlight one way that adversity in otherwise positive social relationships can alter gene expression to impact health. Additionally, relationships can become a source of gene-altering social threat when they involve ongoing interpersonal conflict, violence, or abuse. For example, children who have experienced physical maltreatment exhibit persistently altered gene methylation that impacts expression of the glucocorticoid receptor gene, which can in turn affect inflammation and health.^125^

Other less severe forms of social–environmental adversity—particularly when experienced during childhood, an especially sensitive period of development, or when experienced chronically—have been found to exert long-lasting effects on the expression of glucocorticoid receptor genes, which can result in glucocorticoid insensitivity.^19,21,124^ In a typical stress response, glucocorticoid receptors downregulate inflammatory gene expression. However, in persons who have experienced chronic or early life stress, glucocorticoid receptors often fail to regulate glucocorticoid response genes, despite glucocorticoid levels being either normal, or in many cases, elevated.^59,67,130^ Mechanistically, this process involves *β*AR-induced increases in the production of immature leukocytes from bone marrow that possess desensitized glucocorticoid receptor proteins.^53,59,131,132^ Due to downregulatedglucocorticoid receptor crossregulation, NF-*κ*B and AP-1 bind more readily to gene promoters, in turn upregulating the pro-inflammatory component of the CTRA, leading to heightened inflammatory activity that is not sensitive to the typical anti-inflammatory effects of glucocorticoids.^24,59,133^ Concurrently, sympathetic nervous system/*β*AR signaling also inhibits IFN response factor transcription factors, which suppress antiviral immune response gene expression, increasing a person’s susceptibility to viral infection.^56,67,99,100^

Beyond increases in inflammatory activity and susceptibility to disease, individuals with stress-induced glucocorticoid insensitivity also experience worsening of pre-existing inflammatory diseases, such as asthma and depression,^134,135^ and decreased effectiveness of common glucocorticoid-based treatments for these diseases. On the positive side, research has found that positive social–environmental conditions can improve outcomes. For example, children with asthma who had supportive versus unsupportive parents have been shown to exhibit reduced stress-induced glucocorticoid insensitivity.^115^ Related research has found that maternal warmth can buffer children from the negative impact of early life adversity on adult pro-inflammatory gene expression.^116^ This work demonstrates that although negative social experiences have strong gene-altering effects, positive experiences can buffer individuals from the often health-harming, transcriptome-altering impacts of social adversity.

## 9 | PSYCHOSOCIAL INTERVENTIONS, PROSOCIAL BEHAVIOR, AND GENE EXPRESSION

The literature reviewed above documents how adverse social experiences can negatively affect gene expression. However, there is also emerging evidence that psychosocial interventions can have beneficial effects on gene expression^136^ as well as immunological markers that are reflective of changes in gene expression, such as changes in pro- and anti-inflammatory cytokine levels, antibody levels, and natural killer cell activity.^137^ For example, participating in a 10-week cognitive-behavior therapy (CBT) program focused on managing anxiety and negative cognitions altered gene expression in breast cancer patients who were randomly assigned to receive the intervention.^110,138^ Although stress and anxiety inherent in managing breast cancer would typically result in the CTRA, participants in the CBT intervention instead exhibited reduced pro-inflammatory and metastasis-related gene expression, and increased interferon-related gene expression, providing strong evidence that psychosocial interventions targeting cognitive processes can impact gene expression. Similar genome-regulating effects have been found for the Kirtan Kriya meditation program,^112^ a stress management psychoeducation program,^113^ CBT for insomnia,^139^ and mindfulness-based stress-reduction^111,140,141^ (see also Ref. 142). These effects are hypothesized to occur by altering individuals’ actual social–environmental circumstances, their perceptions of such circumstances, or both.

Psychosocial interventions such as these are poised to alter gene expression in individuals currently facing stress and adversity, such as those providing care for stressed family members or who are facing their own stressful circumstances. One limitation of these interventions, however, is that they are relatively expensive as well as time and labor intensive, making them difficult to deploy on a large scale. This is unfortunate, given the enormous amount of chronic disease burden that is evident worldwide. To more effectively translate knowledge from social genomics research to reduce the impact of stress on disease burden, researchers will need to focus on developing interventions that are effective but also highly scalable.

Emerging evidence suggests that prosocial behavior can also alter gene expression in promising ways. For example, engaging in a volun teering intervention program has been associated with reduced CTRA gene expression and improved self-reported well-being in older adults who volunteered in children’s classrooms.^114^ More generally, positive social relationships may help prevent stress from affecting the basal transcriptome, especially if they are characterized by high levels of social safety.^27,92^ Recent research using the shelter-in-place restrictions enacted during the global coronavirus pandemic as a natural experiment of social isolation found that whereas in-person social connection was associated with reduced CTRA expression, online social connection was not.^118^ Therefore, purely online interventions for fostering social connectedness may be less effective than in-person programs when it comes to changing health-damaging gene expression.

## 10 | TOPICS FOR FURTHER CLARIFICATION AND RESEARCH

Much has been learned in the past two decades regarding the social signal transduction pathways that enable social environments to influence human genome function, as well as the general types of genes that are affected by such dynamics, especially in cells of the immune system. However, the scope of human social genomics extends far beyond what has already been discovered, and several important topics remain to be explored. For example, we now know a great deal about how social factors affect the easily accessible tissue of blood but much less about how such factors impact other tissues that are important for human health (e.g., diseased tissues such as tumors, wounds, etc.), behavior (e.g., brain and muscle tissues), and genome evolution (e.g., reproductive tissues). Research “beyond blood” is thus an important frontier for human social genomics research.

In addition, although much has been learned about how adverse life events could potentially exert persistent effects on genome regulation that last long after a stressor has passed, there is very little experimental research that has intervened in social signal transduction pathways to identify the specific causal mechanisms involved. Indeed, most of the existing research on this topic is fundamentally correlational (even in animal models of epigenetics) and thus cannot definitively identify the causal pathways that would need to be targeted in protective or preventive interventions. Research on effective, efficient, and scalable interventions to block adverse effects is also in its infancy, and much remains to be learned regarding how durable or transient the effects of such positive interventions are on gene regulation and health. Indeed, much of the social genomics literature is focused on genome function as an outcome, and there is much more limited information about the health significance of the genomic effects that have been observed (although several studies have linked CTRA gene expression to disease outcomes such as viral infection, cancer, and cardiovascular atherosclerosis, as well as chronic disease and mortality, see Ref. 61). Additionally, although the CTRA itself has received much research attention and is now mechanistically well-defined in terms of gene regulation by the sympathetic nervous system, less is known about which other human genomic systems might be subject to social regulation or which biological signaling pathways might mediate those effects.

At the broadest theoretical level, social regulation of gene expression implies that human genomes are not just endogenously regulated, or regulated by their fixed environments, but are also subject to some degree of regulatory control by the dynamic social networks in which an individual is embedded. Networks of mutually influencing elements can produce complex “dynamic systems” with unexpected “emergent properties” and regulatory influences that are not evident when elements are examined in isolation. It is likely that such network-level emergent effects operate in stabilizing or shaping human genome function, but no research has yet assessed multiple human genomes as they interact with one another and their broader familial, social, and cultural networks. Moreover, few studies have examined social regulation of genome function in nondeveloped settings, or in contexts with lifestyle or pathogen exposures that are characteristic of the human “environment of evolutionary adaptation”. Finally, most existing research on human social genomic is rather coarse in its timescale and intensity (e.g., cross-sectional or pre–post repeated measures studies), with few longitudinal or high-density, intra-individual studies (e.g., daily-to-weekly measurements over several months or years) having been conducted to date. Greater insights into social regulation of human gene expression will undoubtedly emerge, but stronger study designs using more high-frequency assessment protocols are required.

## 11 | SOCIAL GENOMICS IN HEALTHCARE

**FIGURE 3:**
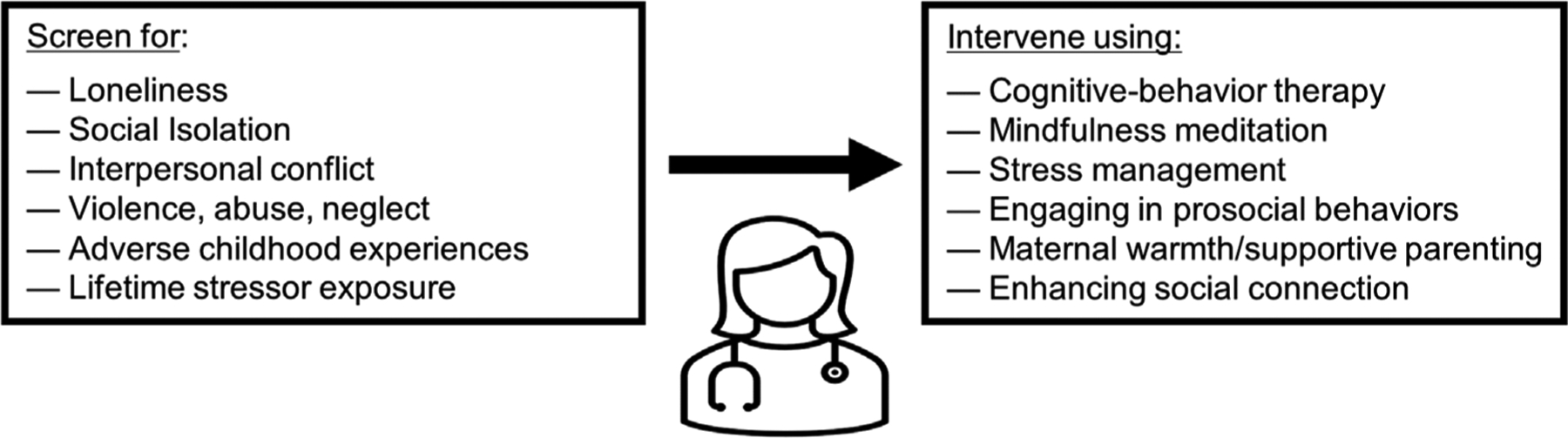
Social genomics in healthcare. To reduce patients’ risk for negative health and behavioral outcomes, health care providers can screen for and respond to factors that have been associated with the Conserved Transcriptional Response to Adversity (CTRA). These factors include loneliness, social isolation, interpersonal conflict, violence, abuse, neglect, adverse childhood experiences, and lifetime stressor exposure. Responding to these factors can involve engaging in trauma- and resilience-informed care, and prescribing evidence-based interventions that can reduce the CTRA, such as cognitive-behavior therapy, mindfulness meditation, stress management, engaging in prosocial behaviors, maternal warmth/supportive parenting programs, and enhancing in-person social connection.

Although much of the research investigating human social geonomics highlights how social adversity can lead to poor health outcomes, understanding social genomics also provides promising strategies by which health care providers may be able to better diagnose, treat, and possibly prevent disease. First, understanding that social– environmental conditions impact gene expression and health gives providers the knowledge needed to identify those at the greatest risk of developing inflammation-related diseases and viral infections. One low-cost, scalable way to leverage this for good is for providers to screen patients for risk factors such as loneliness, social isolation, interpersonal conflict, violence, abuse, and neglect—all of which have been found to alter gene expression—and to then provide stress reduction and resilience-building resources that can improve biological functioning and health (Figure 3). As described above, interventions that alter gene expression and benefit health include CBT, mindfulness meditation, stress management, and engaging in prosocial behaviors, among others.

Second, as humans, we have the remarkable ability to shape our gene expression through our thoughts and beliefs, which may prove to be a surprisingly useful tool if embraced by health care providers in the future. Indeed, although many social–environmental stressors such as chronic unemployment and living in a low-income neighborhood cannot be easily addressed, how people perceive these experiences is critical for shaping health and, importantly, amenable to intervention.^143^ Interventions that modulate patients’ social safety- and threat-related attention, memory, and appraisals may thus be beneficial for enhancing genomic well-being.

Finally, research in human social genomics underscores the importance of screening for major life stressors as well as resilience factors that can affect whether individuals exhibit the CTRA. Screening for adverse childhood experiences (ACEs) and lifetime stressor exposure is becoming more common due to the development of better stress assessment instruments,^144^ but stressor assessments are still not performed in most clinics even though there is good evidence that ACEs impact not just human gene expression but a variety of risk factors for poor health, including health behaviors and engaging in preventative care practices.^145^ As we have discussed herein, early life stress can impact expression of glucocorticoid receptor genes and lead to glucocorticoid insensitivity and greater inflammatory activity. Therefore, screening for and mitigating the negative effects of ACEs on health-relevant transcriptional dynamics should be a top priority that could yield benefits long after an adversity has passed.

## 12 | CONCLUSION

In conclusion, research on human social genomics has begun to elucidate how positive and negative social–environmental experiences get “under the skin” and “onto the genome” to affect human health and behavior. This extant work has identified some of the specific genes and gene programs that can be influenced by the social environment, the biological pathways through which social–environmental experiences impact gene expression, and the genetic polymorphisms that determine the likelihood that physiologic and biochemical signals get fully transduced to ultimately affect gene expression. This research has also pointed to psychosocial interventions and prosocial behaviors that may lead to biologically healthier gene expression profiles. To leverage what is known about human social genomics to better improve the prevention, diagnosis, and treatment of disease, additional research is needed to develop low-cost, effective, and scalable treatments that have the ability to improve human health through impacting gene expression.

## Figures and Tables

**TABLE 1:** Summary of social–environmental factors and interventions that have been associated with changes in human gene expression

Summary of results	Factors/interventions	Selected references
Beneficial influences		
Psychosocial interventions, social engagement and connectedness, and nurturing relationships with parents have been found to decrease expression of pro-inflammatory immune response genes (e.g., *IL1B, IL6,* and *TNF*) and increase expression of antiviral immune response genes (e.g., *IFNA* and *IFNB*), leading to less inflammatory activity and potentially better health outcomes when battling viral threats.	Cognitive-behavior therapy	^110^
	Mindfulness programs	^111^
	Stress management programs	^112,113^
	Volunteering/prosocial behavior	^114^
	Maternal warmth/supportive parenting	^115–117^
	Social connectedness	^118^
Adverse influences		
Adverse social–environmental experiences, including chronic stress, loneliness, poverty, abuse, and early life stress, have been found to increase expression of pro-inflammatory immune response genes (e.g., *IL1B, IL6,* and *TNF)* and decrease expression of antiviral immune response genes (e.g., *IFNA* and *IFNB*), leading to greater inflammatory activity, increased risk of inflammation-related diseases, and worse outcomes when battling viral threats. Further, chronic and early life stress both modify glucocorticoid receptor gene expression in ways that can lead to glucocorticoid insensitivity and systemic chronic inflammation.	Social adversity/chronic stress	^53,76,78,103,119^
	Loneliness and isolation	^24,47^
	Chronic interpersonal stress	^120^
	Abuse/interpersonal violence	^121^
	Rejection	^122,123^
	Caregiving	^67,124^
	Early life stress/ACEs	^21,125,126^

Abbreviations: ACEs, adverse childhood experiences; *IFNA*, interferon alpha; *IFNB*, interferon beta; *IL1B*, interleukin 1 beta; *IL6*, interleukin 6; *TNF*, tumor necrosis factor.
